# Extraction, modification, and property characterization of dietary fiber from *Agrocybe cylindracea*

**DOI:** 10.1002/fsn3.1905

**Published:** 2020-09-27

**Authors:** Fengjuan Jia, Xuecheng Liu, Zhiqing Gong, Wenjia Cui, Yansheng Wang, Wenliang Wang

**Affiliations:** ^1^ Institute of Agro‐Food Science and Technology Shandong Academy of Agricultural Sciences Jinan Shandong PR China; ^2^ Key Laboratory of Agro‐Products Processing Technology of Shandong Province Jinan Shandong PR China; ^3^ Key Laboratory of Novel Food Resources Processing Ministry of Agriculture and Rural Affairs Jinan Shandong PR China

**Keywords:** *Agrocybe cylindracea*, antioxidant property, dietary fiber, extraction, modification

## Abstract

Dietary fiber (DF) has gained a great attention owing to its potential health benefits. *Agrocybe cylindracea* is an edible fungus with high protein and low fat contents, which is also an enriched source of DF. However, limited study has been conducted on optimizing the conditions of *A*. *cylindracea*‐derived DF extraction and modification as well as characterizing its properties. In this study, ultrasound‐assisted enzymatic method for DF extraction was optimized as the following conditions: liquid material ratio of 29 ml/g, α‐amylase concentration of 1.50%, protamex concentration of 1.20%, and ultrasonic power of 150 W, which improved the DF extraction yield to 37.70%. Moreover, high temperature modification (HTM) and cellulase modification (CEM) were applied to modify *A*. *cylindracea*‐derived DF. The results showed that HTM had more potential capacity in converting insoluble DF into soluble DF, and DF with HTM exhibited more advantages in its physicochemical properties than DF with CEM. The DF with both HTM and CEM showed antioxidant activities, reflected by the increased reducing power as well as DPPH radical, hydroxyl radical, and ABTS^+^ scavenging capabilities in vitro. These findings could offer a reference for the extraction, modification, and characterizing various properties of DF from *A*. *cylindracea*, which would establish the foundation for the comprehensive application of fungi‐derived DF.

## Graphical abstract

The dietary fiber (DF) extraction yield from *Agrocybe cylindracea* achieved 37.70%. High temperature‐modified DF exhibited advantages on physicochemical properties. Both high temperature‐ and cellulose‐modified DF exhibited antioxidant activity.

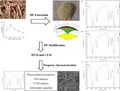

## 1 INTRODUCTION

*Agrocybe cylindracea* have been consumed by a significantly increased number of consumers recently, due to its high values of nutrition and delicious taste. As an edible and medicinal fungi which belongs to basidiomycete, a large number of bioactive components, including glucose, amino acids, proteins, and polysaccharides, have been identified and isolated from the fruiting bodies of *A. cylindracea* (Kim et al., 2005; Liu et al., 2016; Zhao et al., 2017). These components have antitumor, antimutagenic, hypoglycemic, antifungal, neurotonic, antilipid peroxidation, antihypercholesterolemia, immune regulation, antiaging, and antioxidant activities (Hu et al., 2011; Ngai et al., 2003, 2005; Shon & Nam, 2004). However, compared with the polysaccharides and peptides in *A. cylindracea*, the rich content of dietary fiber (DF) is rarely mentioned and has not been effectively utilized.

DF is an indigestible carbohydrate that could not be absorbed by the digestive tract. In recent years, DF has received more attentions for its potential health benefits, such as improving laxation, reducing the risk of cardiovascular disease, enhancing weight management, strengthening immune functions, and promoting colonic health (Granado‐Lorencio & Hernandez‐Alvarez, 2016; Trompette et al., 2018). Based on its various advantages, such as water retention, oil retention, and structural properties, DF is often used as food additives for improving certain properties of the food products (Baye et al., 2017; Ma et al., 2015). It can also extend the shelf life of food products with rich fat contents by increasing the antioxidant capacity of the emulsion (Elleuch et al., 2011). Moreover, multiple biological, chemical, and physical approaches have also been developed to modify food‐derived DFs in terms of their microstructure and composition, for the purpose of improving their physiological and functional properties and applying them in food industry (Khan et al., 2018; Nyman & Svanberg, 2002).

DF is commonly divided into two categories according to its water solubility, namely soluble dietary fiber (SDF) and insoluble dietary fiber (IDF) (Bader Ul Ain et al., 2019). SDF is more important for its higher biofunctions and physiological perspectives than IDF, due to its rapid fermentation, breakdown into short‐chain fatty acids, and higher consumption efficiency by probiotics (Bader Ul Ain et al., 2019). SDF could also bind with cholesterol and sugar to reduce their absorption and transfer in plasma (Abutair et al., 2018; McRae, 2018). Moreover, SDF enters the intestinal tract and softens the stool, preventing constipation and hemorrhoids (Slavin, 2013). Hence, the important physical and chemical properties of DF are closely related to its SDF content.

Various raw materials have been used to extract DF, such as barley grains, coconut kernel, deoiled cumin, rice bran, and soya meal (Abdul‐Hamid, 2000; Ma & Mu, 2016a; Ma & Mu, 2016b; Redgwell et al., 2003). Enzymatic, chemical, and combined approaches are usually applied for the extraction of DF (Jia et al., 2019). For example, acidic treatment on rice bran‐derived DF increased its crystallinity, porosity, and capacity of oil holding (Eroshkin et al., 1987). Wheat bran‐derived DF with ultrasonic and microwave treatments relatively improved its tensile properties and texture to be used for noodles and sensory characteristics (Wang et al., 2018). In our previous study, in comparison with SDF, the content of IDF was significantly higher in *A. cylindracea* (data not published). Therefore, efficient DF extraction from *A*. *cylindracea* and IDF modification into SDF has recently been widely studied.

In this study, the response surface methodology (RSM) was utilized to determine the optimal conditions of DF extraction from *A*. *cylindracea* by ultrasonic‐assisted enzymatic method, which was shown as liquid material ratio at 29 ml/g, 1.50% α‐amylase, 1.20% protamex, and ultrasonic power at 150 W, reaching a DF extraction rate of 37.7%. Furthermore, two modification methods, namely high temperature modification (HTM) and cellulase modification (CEM), were applied for improving the properties of DF. Compared to CEM, HTM could effectively increase the content of SDF. In addition, the antioxidant activities of DF were evaluated following standard procedures, the result of which revealed the advantages of HTM on *A*. *cylindracea*‐derived DF modification.

## 2 MATERIALS AND METHODS

### 2.1 DF extraction from *A. cylindracea*

The *A*. *cylindracea* powder (2 g) was mixed with water at various ratios, adjusted to pH 7.0, hydrolyzed by α‐amylase for 2 hr at 55°C in ultrasonic instrument. The sample was then hydrolyzed by compound protease at 55°C for 2 hr in ultrasonic instrument. Then, the sample was precipitated with 95% alcohol (4:1 w/v) for DF yield. The precipitates were washed with acetone by three times and oven‐dried at 60°C for DF yield. The yield of DF (%) was measured by Equation 1:

(1) $$\text{DF}\left(\%\right)=\text{residualweight}/\text{dryweightof}A.\;cylindracea\text{powder}\times 100\%$$

### 2.2 Optimizing conditions for DF extraction

A Box–Behnken design (BBD) with four independent variables was used for optimizing the conditions for DF extraction. Table S1 lists the independent variables, their ranges, and horizontal levels. The yield of DF (%) was in response to the independent variables (Table 1). The variables were calculated by the following equation:

(2) $$x_{i}=\frac{\left(X_{i}‐X_{0}\right)}{\Delta X_{i}},\;i=1,2,3,\ldots ,k$$

where *x_i_* and *X_i_* are the coded and actual values of the independent variables; *X*_0_ represents the actual value of the independent variable at the center point; and Δ*X_i_* represents the value of step change.

**TABLE 1:** RSM design scheme and DF yield

No.	(A) Liquid material ratio ml/g	(B) α‐amylase concentration %	(C) Protamex concentration %	(D) Ultrasonic power W	DF yield %
1	30	1.5	1.6	200	34.2
2	30	1.5	0.8	100	33.9
3	25	1.0	1.2	150	34.2
4	30	1.5	1.2	150	37.6
5	35	1.5	1.2	200	32.8
6	30	2.0	1.2	200	33.6
7	25	2.0	1.2	150	35.9
8	25	1.5	1.2	100	34.0
9	30	1.0	1.2	200	34.6
10	30	1.5	1.2	150	37.3
11	25	1.5	0.8	150	34.6
12	30	1.5	1.2	150	37.6
13	35	1.0	1.2	150	33.1
14	35	2.0	1.2	150	32.8
15	30	2.0	0.8	150	33.5
16	30	2.0	1.2	100	34.8
17	30	2.0	1.6	150	35.5
18	30	1.0	1.6	150	33.3
19	30	1.5	1.2	150	38.1
20	25	1.5	1.2	200	36.6
21	30	1.5	0.8	200	34.6
22	25	1.5	1.6	150	34.1
23	30	1.5	1.6	100	35.3
24	35	1.5	1.6	150	34.8
25	30	1.0	1.2	100	35.2
26	35	1.5	1.2	100	35.4
27	30	1.5	1.2	150	38.5
28	30	1.0	0.8	150	34.4
29	35	1.5	0.8	150	33.8

DF, dietary fiber.

To establish the correlation between response variable and the independent variables, a quadratic polynomial equation was used for fitting a quadratic model:

(3) $$Y=\beta_{0}+\sum \beta_{i}x_{i}+\sum \beta_{\mathrm{ii}}x_{i}^{2}+\sum \beta_{\mathrm{ij}}x_{i}x_{j}$$

where *Y* represents the predicted response value; *β*_0_, *β_i_*, *β_ii_*, and *β_ij_* represent intercept, linear, squared, and interaction terms, respectively; and *x_i_* and *x_j_* are the coded levels of independent variables.

### 2.3 *Modification of DF from A*. *cylindracea*

#### 2.3.1 High temperature modification (HTM)

Powdery *A*. *cylindracea* DF (2.0 g) was rinsed in tap water with different ratios and then incubated at different temperature for various time periods. After cooling, the samples were centrifuged for 15 min at 6,000 *g*. Then, the supernatant was retained and mixed with ethyl alcohol (1:4 v/v). The white flocculent was extracted, filtered, and washed with acetone, and the SDF product was freeze‐dried. SDF yield (%) represents the proportion of SDF in total DF.

#### 2.3.2 Cellulase modification (CEM)

Powdery *A*. *cylindracea* DF (2.0 g) was hydrolyzed by cellulase at 55°C for different periods of time. Then, the sample was incubated for 5 min at 95°C to deactivate cellulase. After cooling, the samples were centrifuged for 15 min at 6,000 *g*, followed by retaining the supernatant and mixing with ethyl alcohol (1:4 v/v). The white flocculent was extracted, filtered, and washed with acetone, and the SDF product was freeze‐dried. SDF yield (%) represents the proportion of SDF in total DF.

### 2.4 Water binding capacity (WHC)

WHC (g/g) was assessed in accordance with Jia et al. (2019) as the retained distilled water content, by Equation 4:

(4) $$\text{WHC}=\left(m_{r}‐m_{d}\right)/m_{d}$$

where *m_r_* represents the weight of water residue (g) and *m_d_* represents the dry weight of DF (g), assuming there is no loss of soluble materials.

### 2.5 Swelling capacity (SC)

SC (ml/g) was assessed in accordance with Jia et al. (2019) by Equation 5:

(5) $$\text{SC}=\left(V_{r}‐V_{d}\right)/m_{d}$$

where *V_r_* represents the volume after expansion (ml), *V_d_* represents the dry volume of DF (ml), and m_d_ represents the dry weight of DF (g).

### 2.6 Fat adsorption capacity (FAC)

FAC (g/g) was assessed in accordance with Ma et al. (2015) as the retained sunflower oil, by Equation 6:

(6) $$\text{FAC}=\left(m_{r}‐\;m_{d}\right)\times 100/m_{d}$$

where *m_r_* represents the residual weight of retained oil (g) and m_d_ represents the dry weight of DF (g).

### 2.7 Glucose adsorption capacity (GAC)

GAC (mmol/g) was estimated according to Ma et al. (2015) by Equation 7:

(7) $$\text{GAC}=\left(Ci‐Cs\right)\times Vi/Ws$$

where *Ci* represents the original glucose content (mmol/L), *Cs* represents the supernatant glucose content with equilibrium adsorption (mmol/L), *Ws* represents DF weight (g), and *Vi* represents the supernatant volume of glucose solution (ml).

### 2.8 Cholesterol adsorption capacity (CAC)

The fresh egg yolk was mixed with distilled water (1:9 w/v) to form an emulsion homogenizer. The cholesterol content in the emulsion was determined by phthalaldehyde (W_1_). DF sample (m) was added to the diluted yolk solution (1:25 w/v; g/ml), followed by shaking for 4 hr at room temperature and centrifugation for 15 min at 6,000 *g*. The cholesterol content in the supernatant was also determined (W_2_). CAC (mg/g) was determined using Equation 8:

(8) $$\text{CAC}=\left(W_{1}‐W_{2}\right)/m$$

### 2.9 Cation exchange capacity (CEC)

An aliquot of 1.0 g dry sample was rinsed for 24 hr in 0.1 mol/L hydrochloric acid solution. Then, the excessive acid was removed, and the residue was washed by distilled water for three times, ensuring the solution pH > 4. Then solution was treated with 10% AgNO_3_ titration for removing Cl^‐^, followed by drying the sample and suspending the residue in 50 ml of sodium chloride solution (0.3 M). A blank test was performed with distilled water. After 24 hr continuously shaking, the sample was centrifuged for 10 min at 6,000 *g*, and the supernatant was dripped with 0.01 mol/L sodium hydroxide solution until pH = 7. The amount of sodium hydroxide used for neutralizing supernatant pH was recorded.

### 2.10 Fourier‐transformed infrared spectroscopy (FTIR)

DF was mixed with potassium bromide (KBr) for pellet formation (thickness of 1–2 mm), with the pure KBr served as control. Thermo Nicolet 67 FTIR spectrometer with a DTGS detector was used at 0.09 cm^−1^ resolution in range of 400–4,000 cm^−1^.

### 2.11 Scanning electron microscopy (SEM)

DF was examined with *SEM* (JSM‐6490LV, Japan) at 15 kV. The powder samples were mounted on a metal platform and then sputter‐coated with gold. The images for individual samples were taken at the magnifications of 200, 500, and 1,000×, respectively.

### 2.12 Reducing power

The dry DF powder was mixed with 70% ethyl alcohol (1:50 w/v) and shaken for 2 hr at 60°C. Then, the mixture was centrifuged for 15 min at 4,000 rpm under 20°C, followed by collecting the supernatant for further analysis. The reducing power was assessed based on the description of Zhang (Zhang et al., 2016).

### 2.13 DPPH scavenging assay

The DPPH scavenging assay was estimated following the method described by Xu (Xu et al., 2017) with slight modifications. The absorbance of the mixture containing samples and ethanol (*A*_0_) or DPPH (A) was measured at 517 nm. The scavenging ability was assessed by Equation 9:

(9) $$\text{Scavenging ability}\left(\%\right)=\left(1‐A/A_{0}\right)\times 100\%$$

### 2.14 Hydroxyl radical scavenging capability

The hydroxyl radical scavenging capability was assessed according to Zhang (Zhang et al., 2016) with brief modifications. The absorbance of the samples (A) and the blank (*A*_0_) were measured at 510 nm, and the hydroxyl radical scavenging ability was assessed by Equation 10:

(10) $$\text{Scavenging ability}\left(\%\right)=\left(1‐A/A_{0}\right)\times 100\%$$

### 2.15 ABTS^+^ scavenging ability

The ABTS^+^ scavenging abilities were estimated following the method of Roberta (Re et al., 1999). The absorbance was measured at 734 nm. Anhydrous ethanol served as the blank. The ABTS^+^ removing capability of the sample was reflected by percent decrease of the absorbance. Trolox was used as the reference for creating standard curve. The determined total antioxidant capability was compared with the free radical scavenging capability of Trolox.

### 2.16 Statistical analyses

Significant differences among groups were determined by one‐way analysis of variance (ANOVA) and Duncan's multiple comparison test using SPSS software (Statistical Product and Service Solutions, IBM SPSS Statistic version 17, Armonk, NY, USA). Data were displayed as mean ± standard deviation. A *p*‐value less than .05 indicated statistical significance.

## 3 RESULTS

### 3.1 Single factor effect on DF yield from *A. cylindracea*

**FIGURE 1:**
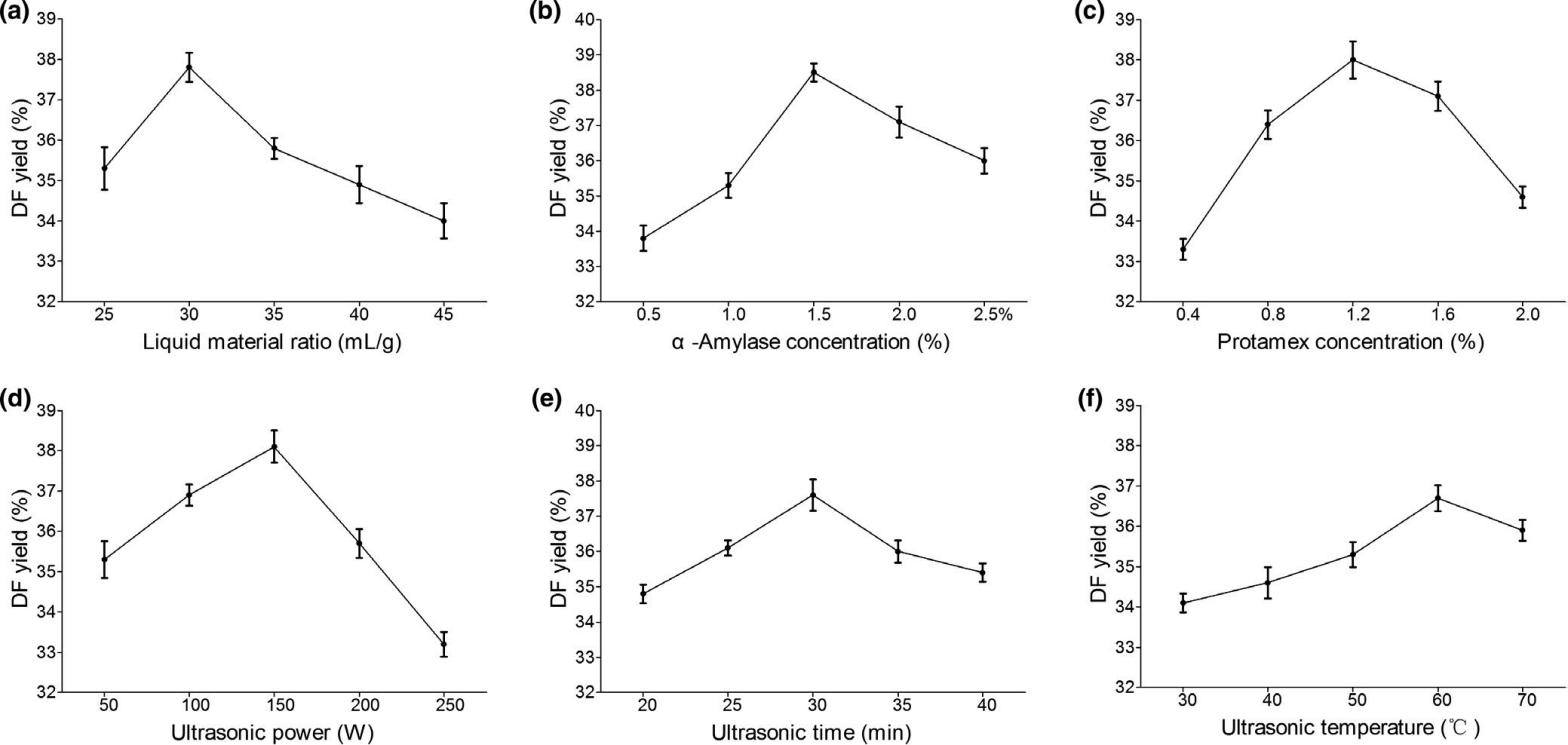
Effects of independent variables on dietary fiber yield from *Agrocybe cylindracea*. The independent variable was examined by keeping other variables constant, based on the default values of liquid material ratio = 30 ml/g, α‐amylase concentration = 1.5%, protamex concentration = 1.2%, ultrasonic power = 150 W, ultrasonic time = 30 min, and ultrasonic temperature = 60°C; (a) liquid material ratio; (b) α‐amylase concentration; (c) protamex concentration; (d) ultrasonic power; (e) ultrasonic time; and (f) ultrasonic temperature

The effects of 6 independent variables, including liquid material ratio, α‐amylase concentration, protamex concentration, ultrasonic power, ultrasonic time, and ultrasonic temperature, on DF yield from *A*. *cylindracea* were determined. The peak DF yields were obtained by liquid material ratio as 45 ml/g (Figure 1a), α‐amylase concentration as 1.5% (Figure 1b), protamex concentration as 1.2% (Figure 1c), ultrasonic power as 150W (Figure 1d), ultrasonic time as 30 min (Figure 1e), or ultrasonic temperature as 60°C (Figure 1f), when the other variables were remained constant. Generally, different variables exhibited similar patterns in affecting the extraction yield of DF, among which ultrasonic time and temperature had relatively less influences (Figure 1e and f).

### 3.2 Model fitting and statistical analyses

On the basis of the effect of single factor on DF yield, the variables further considered for RSM were liquid material ratio (A), α‐amylase concentration (B), protamex concentration (C), and ultrasonic power (D). As shown in Table S1, 30 ml/g liquid material ratio, 1.5% α‐amylase, 1.2% protamex, and 150 W ultrasonic power were selected for the central composite design to the optimization of DF extraction. The RSM design scheme and corresponding DF yield are shown in Table 1. The highest yield of DF (38.5%, No. 27) was achieved by 30 ml/g liquid material ratio, 1.5% α‐amylase, 1.2% protamex, and 150 W ultrasonic power. RSM was applied to assess the quadratic model fitting for DF yield following Equation 11:

(11) $$\begin{array}{c} Y=37.82‐0.56A+0.11B+0.2C‐0.18D\\ \;\;\;\;‐0.15\text{AB}\;+0.38\text{AC}‐1.3\text{AD}+0.78\text{BC}‐0.15\text{BD}\\ \;\;\;\;‐0.45\text{CD}‐1.77A^{2}‐1.92B^{2}‐1.79C^{2}‐1.41D^{2} \end{array}$$

The regression model *Y* was highly significant (*p* < .01), while the lack of fit was not significant (*p* = .3039). The linear term regression coefficient (A) (*p* = .0058), interaction coefficients (AD and BC), and quadratic coefficients (A^2^, B^2^, C^2^, and D^2^) were significant (*p* < .0001), indicating that liquid material ratio, concentrations of α‐amylase and protamex, and ultrasonic power were all significantly correlated with DF yield. Moreover, the impacts of the four variables on DF yield were ranked (from the highest to the lowest) as follows: A, C, D, and B. The determination coefficient (R^2^) of the predicted model was 0.9309, indicating that the experimental values were well aligned with the predicted values (Table 2).

### 3.3 Optimizing parameters for DF extraction through RSM

**FIGURE 2:**
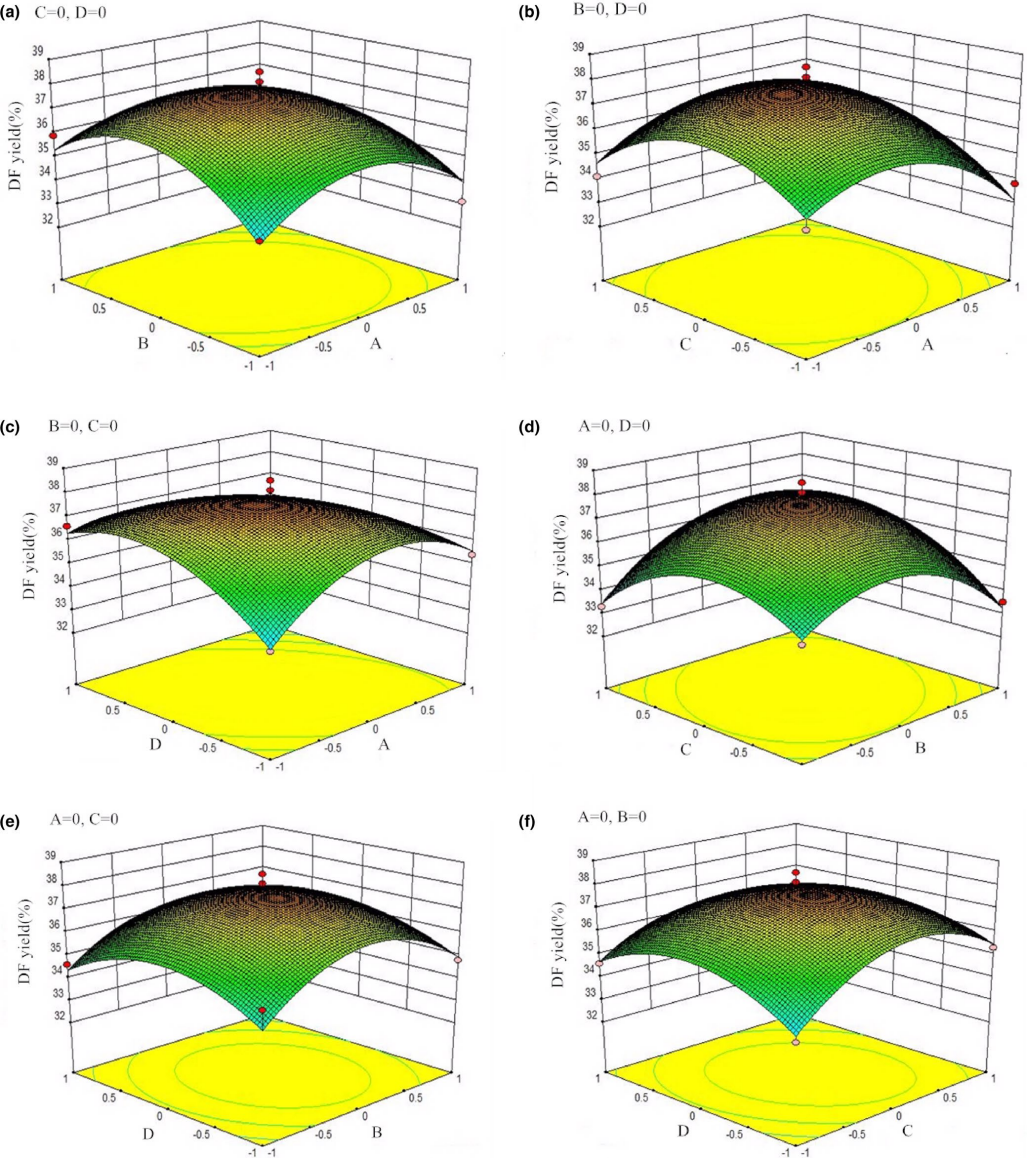
Response surface plots for independent variables on dietary fiber extraction yield from *Agrocybe cylindracea*. (a) Liquid material ratio; (b) concentration of α‐amylase; (c) concentration of protamex; and (d) ultrasonic power

The optimum extraction conditions were optimized by canonical analysis, and the relationships among responses and processing variable levels, as well as variable interactions, were visualized by generating tridimensional response surface plots. As shown in Figure 2, two continuous variables were established for the extraction rate of DF, while the others constant at the zero level were considered as the central value in testing range. The increased interaction values (AB, AC, AD, BC, BD, and CD) before reaching the peaks could indicate an increasing extraction rate of DF, while the increased parameter values afterward indicate lowered extraction rate of DF. Based on the equation 11 using RSM, the optimal extraction conditions to obtain maximum yield of DF were 29.20 ml/g liquid material ratio, 1.53% α‐amylase, 1.22% protamex, and 149.86 W ultrasonic power, in obtaining the maximum DF yield at 37.87%. Considering the convenience in processing, the following extraction parameters were determined: liquid material ratio at 29 ml/g, α‐amylase concentration at 1.50%, protamex concentration at 1.20%, and ultrasonic power at 150 W. Under the abovementioned conditions, the DF extraction yield achieved 37.70%, with no significant difference compared with the theoretical prediction.

**TABLE 2:** Analysis of variance (ANOVA)

Source	Sum of squares	Degree of freedom	Mean square	*F*‐value	*p*‐value	Significance analysis
Model	66.65	14	4.76	13.46	<.0001	**
A	3.74	1	3.74	10.58	.0058	**
B	0.14	1	0.14	0.4	.5381	
C	0.48	1	0.48	1.36	.2635	
D	0.4	1	0.4	1.14	.3036	
AB	1	1	1	2.83	.1148	
AC	0.56	1	0.56	1.59	.2278	
AD	6.76	1	6.76	19.12	.0006	**
BC	2.4	1	2.4	6.79	.0207	*
BD	0.09	1	0.09	0.25	.6218	
CD	0.81	1	0.81	2.29	.1524	
A^2^	20.38	1	20.38	57.63	<.0001	**
B^2^	23.97	1	23.97	67.8	<.0001	**
C^2^	20.67	1	20.67	58.45	<.0001	**
D^2^	12.9	1	12.9	36.47	<.0001	**
Total error	4.95	14	0.35			
Lack of fit	4.04	10	0.4	1.78	.3039	
Pure error	0.91	4	0.23			
Cor total	71.6	28				
Predicted *R* ^2^ =.9309						

### 3.4 *Modification of DF from A*. *cylindracea*

**FIGURE 3:**
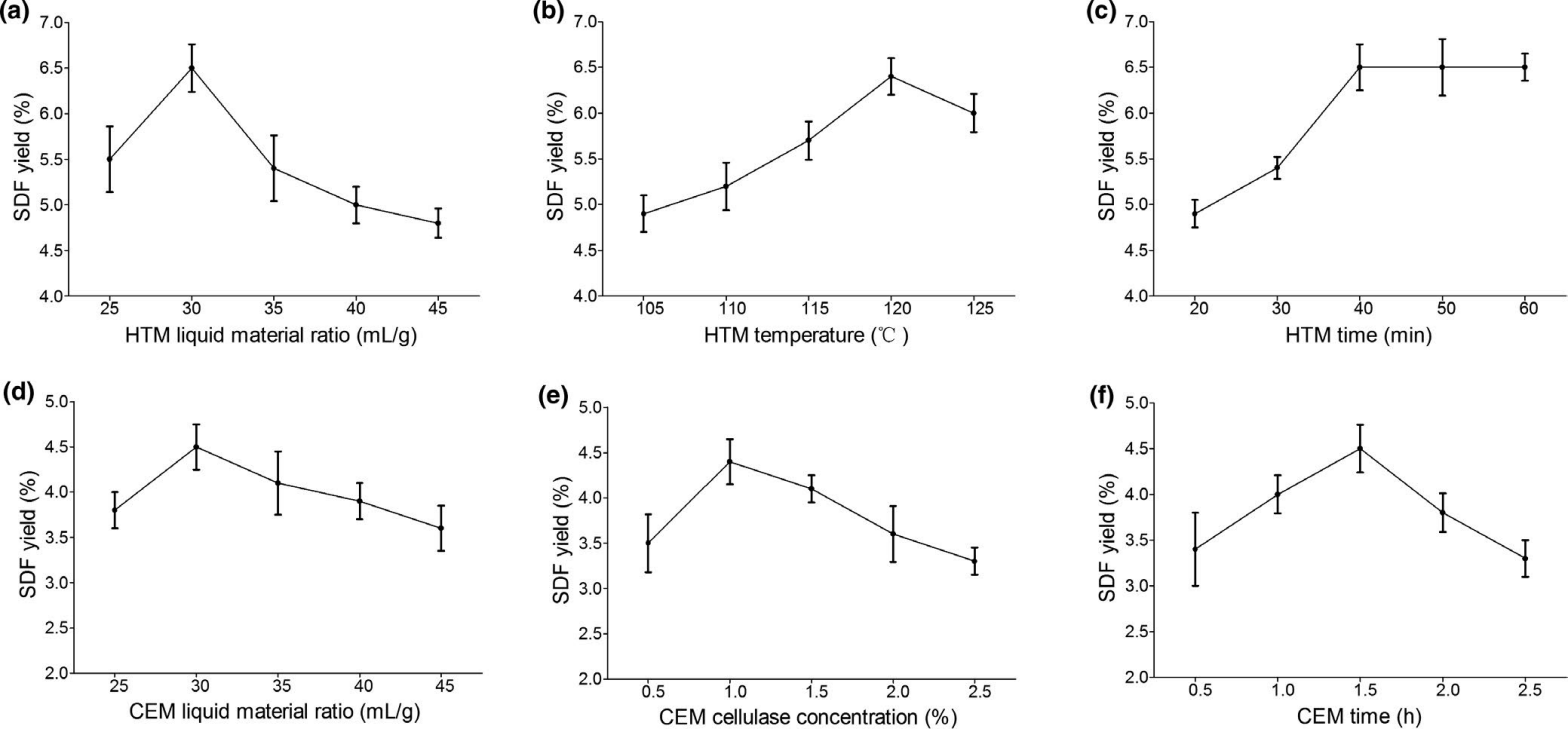
Effects of independent variables on the dietary fiber yield for high temperature modification (HTM) and cellulase modification (CEM). The variables for HTM were examined by keeping other variables constant, based on the default values of liquid material ratio = 30 ml/g, temperature = 120°C, and time = 40 min; (a) HTM liquid material ratio; (b) HTM temperature; and (c) HTM time. The variables for CEM were examined by keeping other variables constant, based on the default values of liquid material ratio = 30 ml/g, cellulase concentration = 1.5%, and time = 2.0 hr; (d) CEM liquid material ratio; (e) CEM cellulase concentration; and (f) CEM time

To further improve the physicochemical properties of *A*. *cylindracea*‐derived DF, two modification methods, HTM and CEM, were used. The mean values for SDF content with HTM are exhibited in Figure 3a–c, and the orthogonal experiment is illustrated in Table S2. The degree of influences on the yield of modified SDF by each factor was HTM time > HTM temperature > HTM liquid material ratio (Table 3). The optimal modification conditions were HTM liquid material ratio of 30 ml/g, HTM temperature of 125°C, and HTM time of 50 min, which contributed to 6.8% yield of SDF. On the other hand, CEM liquid material ratio, CEM cellulase concentration, and CEM time were selected as single factors for orthogonal experiment for modifying *A*. *cylindracea*‐derived DF by CEM (Figure 3d–f, Table S3). The degree of influences on the yield of modified SDF by each factor was CEM cellulase concentration > CEM liquid material ratio > CEM time (Table 4). The optimal modification conditions were CEM liquid material ratio of 30 ml/g, CEM cellulase concentration of 1.5%, and CEM time of 2.0 hr, which contributed to 4.9% yield of SDF.

**TABLE 3:** Orthogonal experimental design and results of HTM

No.	A: HTM liquid material ratio (ml/g)	B: HTM temperature (°C)	C: HTM time (min)	SDF yield (%)	
1	25	115	30	4.2	
2	25	120	40	5.5	
3	25	125	50	6.4	
4	30	115	40	4.8	
5	30	120	50	6.6	
6	30	125	30	5.1	
7	35	115	50	4.9	
8	35	120	30	4.6	
9	35	125	40	5.9	
*k*_1_	5.367	4.633	4.633		
*k*_2_	5.500	5.567	5.400		
*k*_3_	5.133	5.800	5.967		
Range	0.367	1.167	1.334		
Important order	C > B > A			
Optimal combination	A_2_B_3_C_3_			

HTM, high temperature modification; SDF, soluble dietary fiber.

**TABLE 4:** Orthogonal experimental design and results of CEM

No.	A: CEM liquid material ratio (ml/g)	B: CEM cellulase concentration (%)	C: CEM time (hr)	SDF yield (%)
1	25	0.5	1.0	2.8
2	25	1.0	1.5	3.5
3	25	1.5	2.0	4.3
4	30	0.5	1.5	3.4
5	30	1.0	2.0	5.7
6	30	1.5	1.0	4.5
7	35	0.5	2.0	3.2
8	35	1.0	1.0	4.8
9	35	1.5	1.5	5.5
*k*_1_	3.533	3.133	4.033	
*k*_2_	4.533	4.667	4.133	
*k*_3_	4.500	4.767	4.400	
Range	1.000	1.634	0.367	
Important order	B > A>C			
Optimal combination	A_2_B_3_C_3_			

CEM, cellulase modification; SDF, soluble dietary fiber.

### 3.5 *Physicochemical properties of A*. *cylindracea‐derived DF*

**FIGURE 4:**
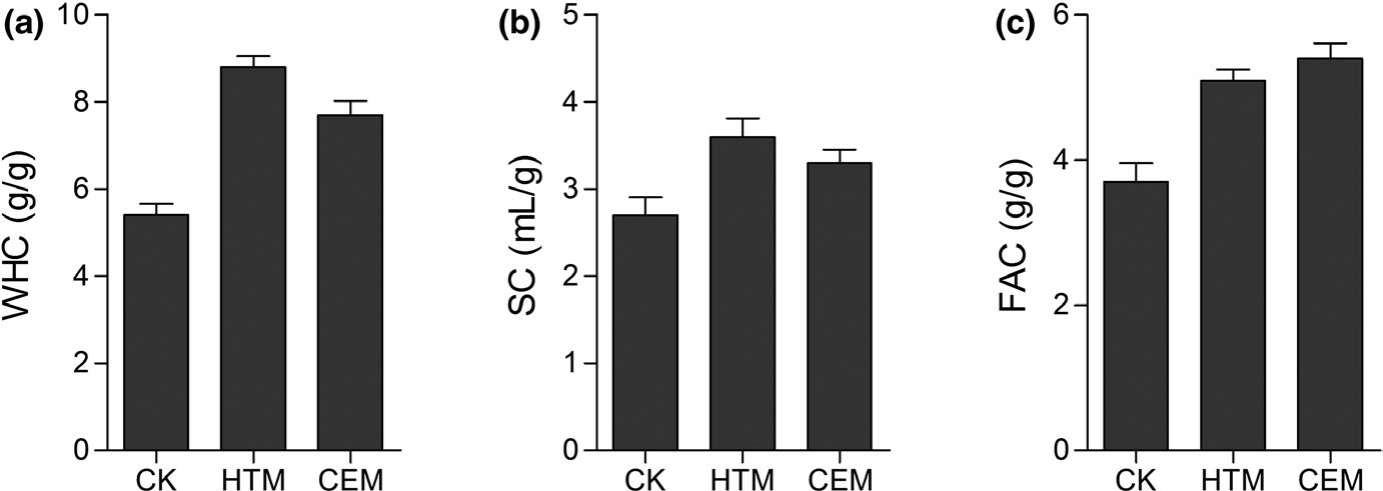
Water binding capacity (a), swelling capacity (b), and fat adsorption capacity (c) of dietary fiber with different modifications

To compare the advantages of different modification methods on DF properties, the physicochemical properties of *A. cylindracea*‐derived DF, including WHC, SC, and FAC, were assessed. After HTM and CEM, the WHC of DF was significantly increased, and HTM exhibited an obvious advantage over CEM (Figure 8a). HTM and CEM raised the SC of DF to 3.6 and 3.3 ml/g, respectively (Figure 4b). The FAC of *A*. *cylindracea*‐derived DF was significantly improved by both methods, while CEM exhibited an advantage over HTM (Figure 4c).

**FIGURE 5:**
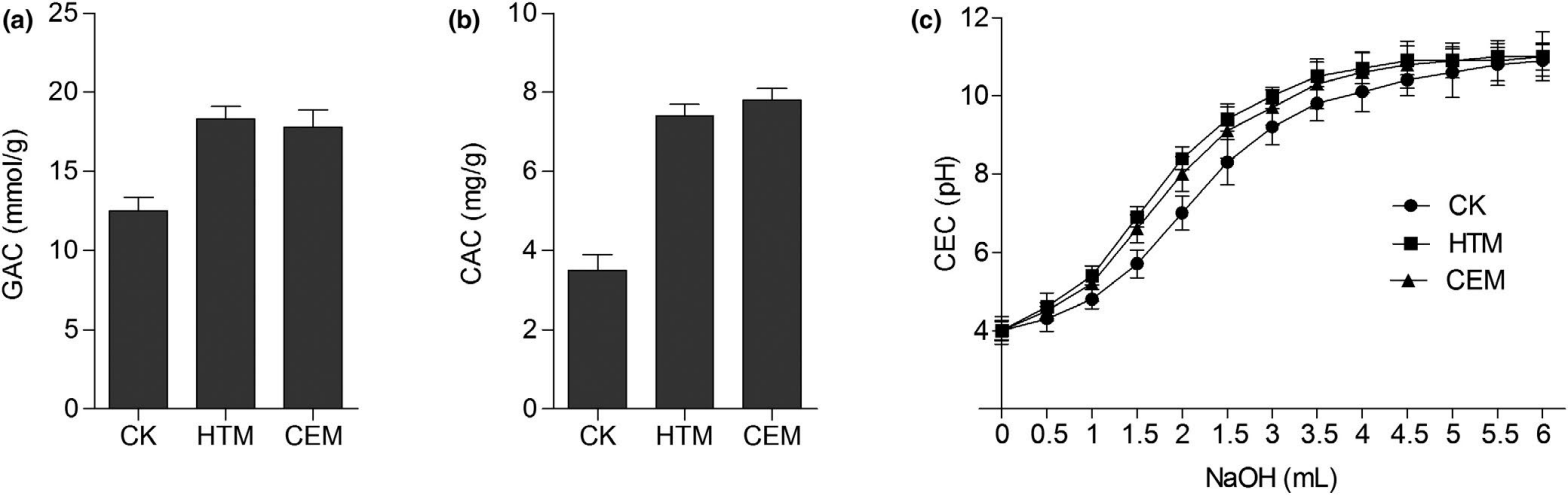
Glucose adsorption capacity (a), cholesterol adsorption capacity (b), and cation exchange capacity (c) of dietary fiber with different modifications

Furthermore, chemical properties including GAC, CAC, and CEC of *A. cylindracea*‐derived DF were also evaluated. Both modification approaches could substantially improve its GAC, with an increase from 12.5 to 17.8 and 18.3 mmol/g, respectively (Figure 5a). HTM and CEM also significantly increased CAC by nearly twofold, corresponding with FAC, while HTM exhibited an advantage on improving CEC (Figure 5c).

### 3.6 SEM analysis of *A. cylindracea*‐derived DF

**FIGURE 6:**
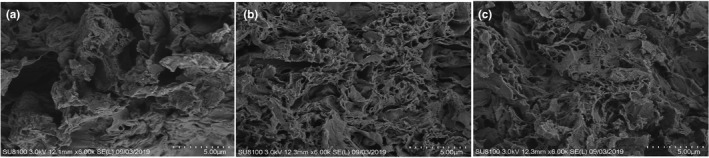
Scanning electron micrographs of dietary fiber (DF) with different modifications. (a) Untreated DF; (b) DF with HTM; and (c) DF with CEM

To further explore the influence of different modification methods on the properties of *A*. *cylindracea*‐derived DF, *SEM* micrographs of DF, DF with HTM, and DF with CEM were performed. The DF structure of the control sample was compact (Figure 6a), while the structure of DF with HTM was loose and contained porous surface, and filiform structures were mostly presented on its surface (Figure 6b). There were more porous structures in DF with CEM (Figure 6c).

### 3.7 *FTIR analysis of A*. *cylindracea‐derived DF*

**FIGURE 7:**
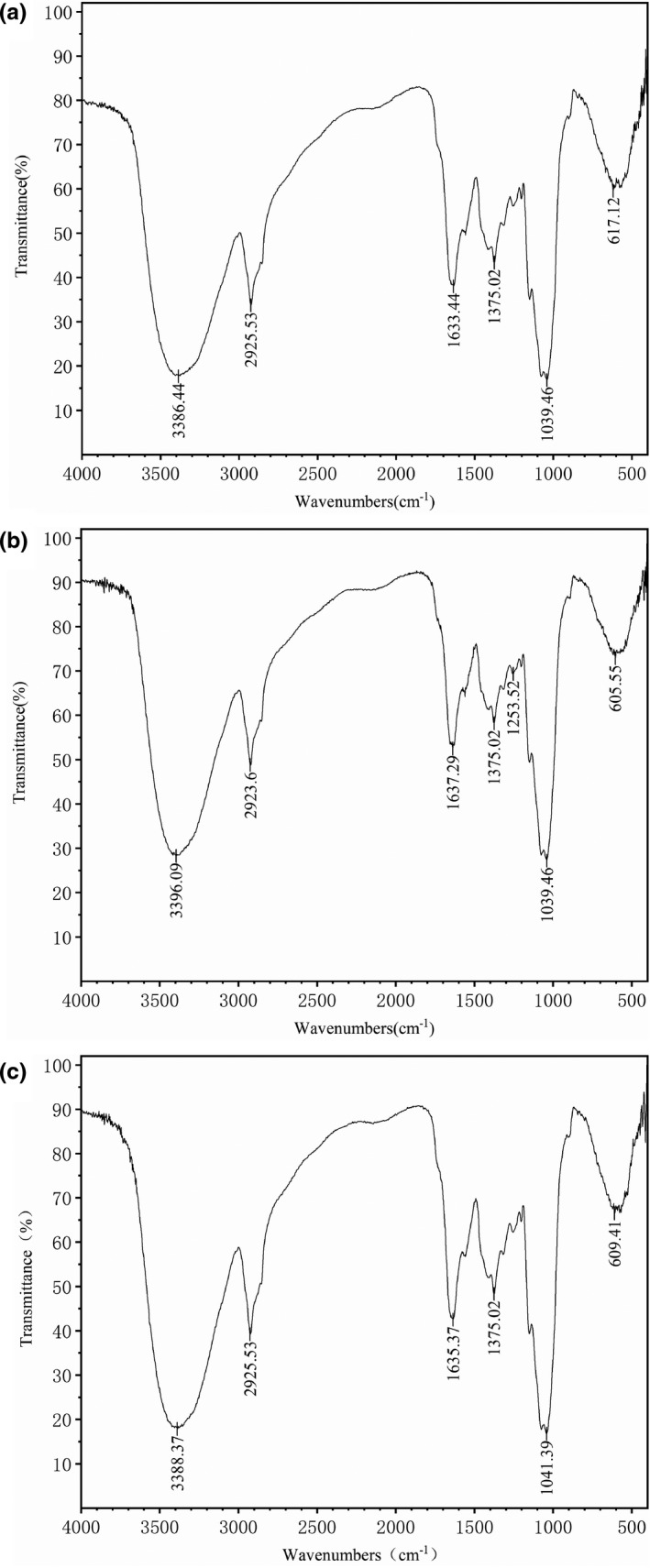
FTIR spectrometric analysis of dietary fiber (DF) by frequency ranging 4000–400 cm^−1^. (a) Untreated DF; (b) DF with HTM; and (c) DF with CEM

FTIR analysis was performed on DFs modified by HTM and CEM to further investigate their structural characterization (Figure 7). We found that DF, DF with HTM or CEM shared similar spectra with little differences observed in wavelength and/or absorbance. The wide band located near 3,400 cm^−1^ was O‐H bound extension to hydrogen and hydroxyl groups, which were generated by hemicelluloses and cellulose, and the absorption peaks were positioned at 3,386.44, 3,396.09, and 3,388.37 cm^−1^ in DF, DF with HTM, and DF with CEM, respectively. The band at 2,920 cm^−1^ was in relevant with C‐H vibration of methylene and methyl, and the absorption peaks were almost the same in different DFs. Moreover, the relatively intensive absorbance peaks at 1,633.44, 1,637.29, and 1,635.37 cm^−1^ in DF, DF with HTM, and DF with CEM, respectively, were caused by asymmetric stretching vibrations of carboxyl group COOH. Another distinctive absorption peak was located at 1,040 cm^−1^, in accordance with characteristic bending or stretching COOH. The peak of DF with HTM was the same with that of the control (1,039.46 cm^−1^), while the peak of DF with CEM was 1,041.39 cm^−1^. Additionally, the C‐H bond absorption peaks of all DFs were positioned at 1,375.02 cm^−1^.

### 3.8 Antioxidant capacities of DF in vitro

**FIGURE 8:**
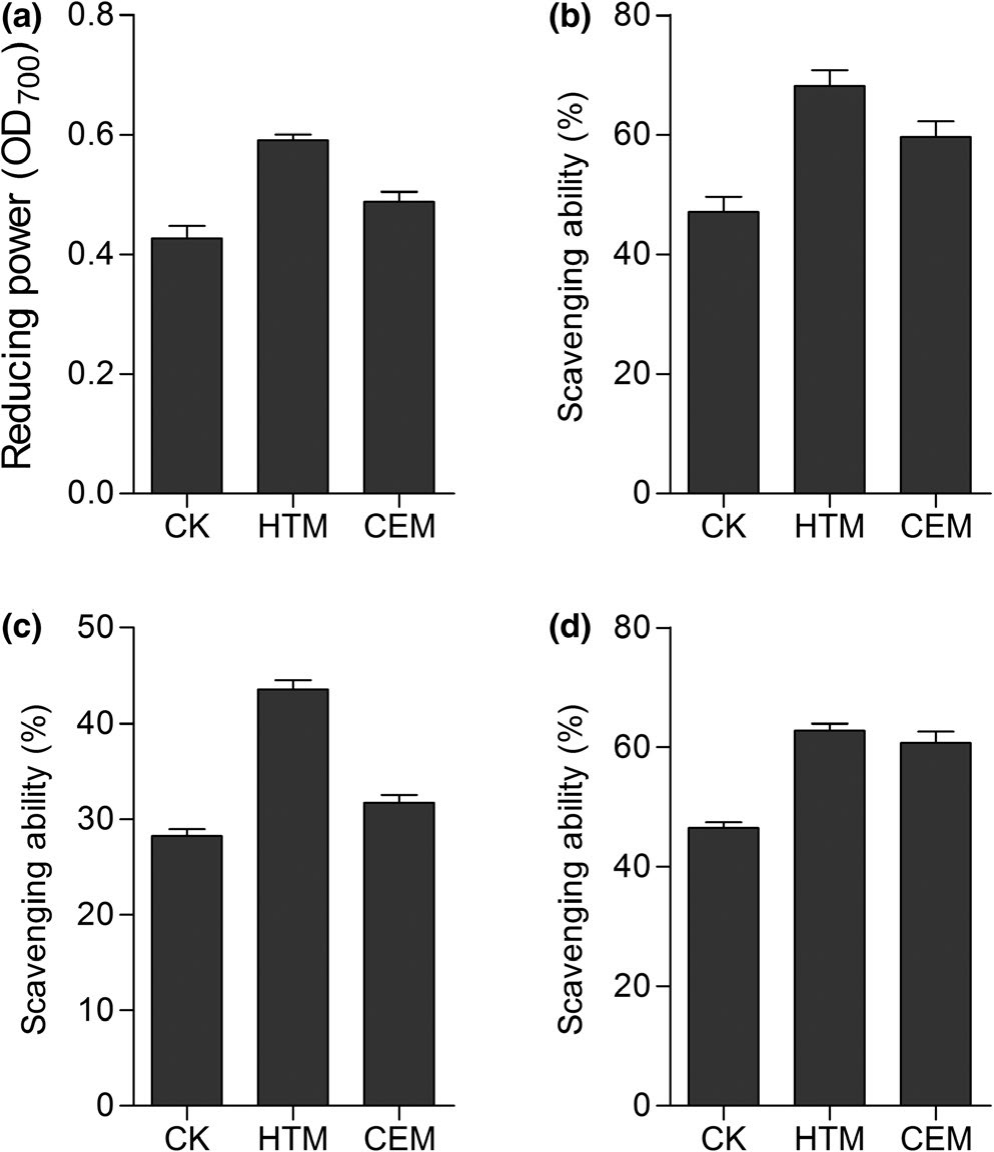
Antioxidant activities of dietary fiber with different modifications in vitro. (a) Reducing power; (b) DPPH radical scavenging activity; (c) hydroxyl radical scavenging activity; and (d) ABTS^+^ scavenging activity

To analyze the antioxidant activities of DFs in vitro, reducing power, DPPH, hydroxyl, and ABTS^+^ scavenging activities were measured. HTM and CEM significantly increased the reducing power of DF (Figure 8a). Moreover, the reducing power of DF with HTM was 22.9% higher than that of DF with CEM. Compared with the control, the modified DFs exhibited stronger DPPH (Figure 8b), hydroxyl (Figure 8c), and ABTS^+^ scavenging activities (Figure 8d), though DF with CEM had a similar hydroxyl scavenging activity as the control sample, indicating the advantage of HTM in improving the antioxidant activities of DF.

## 4 DISCUSSION

As the most crucial edible section in plants, DF remains undigested in the small intestine of human and is completely fermented in the large intestine for its functionality. Currently, several methods have been used to extract DFs from various food sources. During extraction, the conditions of processing can influence the microstructure and composition of DFs, further causing desirable or undesirable effects on their functional and physicochemical properties. For example, due to glycosidic linkage disruption, chemical methods can lead to 100% SDF, 30%–40% hemicelluloses, and 10%–20% cellulose loss during extraction (Nyman and Svanberg, 2002). Moreover, a large quantity of strong alkalis and acids are discarded during industrial processing, leading to serious environmental pollution. Enzymolysis technology has multiple advantages, including easy scale‐up, high production, and mild reaction conditions, over other extraction methods (Yvonne et al., 2016). Furthermore, it can improve extraction system performance and retain extraction composition (Gordon & Okuma, 2002). Additionally, ultrasonic technology has been recently applied for research studies and in food industry, based on its environmentally friendly nature. The interactions between ultrasonic waves and liquid medium or the dissolved gases can cause acoustic cavitation, affecting the structure and morphology of carbohydrate polymers (Minjares‐Fuentes et al., 2016). Moreover, ultrasonic treatments can also reduce the extraction time while increase the reaction rate and extraction yield (Zhang et al., 2017). However, there is little evidence on the extraction of DF from *A*. *cylindracea* through ultrasonic‐assisted enzymatic method. In the current study, RSM was applied to optimize the conditions for *A*. *cylindracea*‐derived DF extraction by ultrasonic‐assisted enzymatic method, focusing on 4 independent variables, including liquid material ratio, concentration of α‐amylase, concentration of protamex, and ultrasonic power. We reveal that enzymolysis extraction combined with ultrasonic method was an effective and efficient method to extract DF from *A*. *cylindracea*.

DF modification strongly influences its functions. IDF is composed of functional groups that efficiently combine with oil, water, or metal ions with toxicity (Elleuch et al., 2011). However, these groups or their binding sites cannot be adequately exposed during classic extraction and hydrolysis. Recently, multiple novel chemical, physical, and biological methods have been employed to modify the microstructure and composition of DFs that are extracted from different foods, to enhance their functional and physiological properties. Previously, deoiled cumin‐derived DF modified by cellulase and laccase with high hydrostatic pressure increased its SDF content and improved its properties (Ma & Mu, 2016a; Ma & Mu, 2016b). Carrot pomace‐derived IDF with complex enzymes, ultrafine comminution, and high hydrostatic pressure treatments positively influenced most of its properties, while ultrafine comminution decreased its water and oil retention capacities (Yu et al., 2018). The current research was carried out to investigate the effect of HTM and CEM on improving the functional properties of *A*. *cylindracea*‐derived DF. It was clear that these two modification methods exerted distinctive effects on improving properties of DF. The WHC, SC, GAC, and CEC of DF by HTM were relatively higher than those of DF by CEM. On the contrary, DF with CEM had comparative advantages on improving FAC and CAC. Compared to DF with CEM, DF with HTM exhibited a honeycomb‐like surface, which might be relevant with the destruction of hemicellulose and cellulose. Moreover, the structural integrity and surface roughness of HTM treated DF were decreased, exhibiting a loose structure as well, which might be responsible for its improved properties and activities. Additionally, the destruction or extension of O‐H, asymmetric stretching vibrations of carboxyl group, and bending or stretching COOH might also contribute to the structural alteration and property improvements DF.

A broad range of oxygen‐free radicals, especially superoxide anion radicals (O_2_^•−^) and DPPH radical, are formed in human bodies and food system (Navarro‐González et al., 2011). Agents capable of eliminating these radicals serve as significant indicators of their antioxidant activity (Dong et al., 2013). Dietary antioxidants can enhance cellular defense and assist in protecting cellular components from oxidative damage (Urszula et al., 2014). Hence, discovering natural compounds with potential antioxidant activities is of great importance for developing treatment on oxidative‐induced diseases. Various DFs have potential antioxidant activities, for example, wheat bran IDF with modifications of complex enzymatic hydrolysis, carboxymethylation, and ultrafine comminution exhibited distinctive antioxidant properties (Zhang et al., 2019). In this study, we showed that *A*. *cylindracea*‐derived DF possessed strong antioxidant activity, and DF with HTM exhibited more intensive antioxidant properties than DF with CEM in vitro (Figure 8). HTM and CEM treatments significantly increased SDF and decreased IDF, which contributed to the significantly promoted antioxidant activities of DF. Overall, these DF modifications established the foundation for the improvement of functional, physiological, and physiochemical properties of DF through elevating its SDF content, as well as for the exploration of more effective and efficient DF modification methods.

In conclusion, heat and enzymatic treatment by various techniques are considered as significant approaches for DF modification, owing to their safety in maintaining the molecular structure of DF and efficiency in increasing the content of SDF. We aimed to extract DF from *A*. *cylindracea* and partially convert IDF into SDF through ultrasonic‐assisted enzymatic method combining with modifications. The present work provides effective modification methods to improve the physicochemical and antioxidant properties of *A*. *cylindracea*‐derived DF in food products, which establishes important theoretical basis for its further comprehensive applications.

## CONFLICT OF INTEREST

The authors declare that they do not have any conflict of interest.

## Supporting information

Table S1‐S3
