# Supplementary material for: Extraction, modification, and property characterization of dietary fiber from Agrocybe cylindracea
Source: Food Sci Nutr. 2020 Sep 27;8(11):6131–43. doi: 10.1002/fsn3.1905 (PMC7684601; doi:10.1002/fsn3.1905)
Supplement: Supplementary file 1 — Table S1‐S3 [file FSN3-8-6131-s001.docx]

Table S1 Box-behnken design scheme

| Horizontal | Liquid material ratio (mL/g) | α-Amylase concentration (%) | Protamex concentration (%) | Ultrasonic power (W) |
| --- | --- | --- | --- | --- |
|  |  |  |  |  |
| -1 | 25 | 1.0 | 0.8 | 100 |
| 0 | 30 | 1.5 | 1.2 | 150 |
| 1 | 35 | 2.0 | 1.6 | 200 |

Table S2 Orthogonal experimental factors of DF modified by HTM

| Levels | Factors | | |
| --- | --- | --- | --- |
|  | A: HTM liquid material ratio (mL/g) | B: HTM temperature  (°C) | C: HTM time  (min) |
| 1 | 25 | 115 | 30 |
| 2 | 30 | 120 | 40 |
| 3 | 35 | 125 | 50 |

DF represents dietary fiber; HTM represents high temperature modification.

Table S3 Orthogonal experimental factors of DF modified by CEM

| Levels | Factors | | |
| --- | --- | --- | --- |
|  | A: CEM liquid material ratio (mL/g) | B: CEM cellulase concentration (%) | C: CEM time  (h) |
| 1 | 25 | 0.5 | 1.0 |
| 2 | 30 | 1.0 | 1.5 |
| 3 | 35 | 1.5 | 2.0 |

DF represents dietary fiber; CEM represents cellulase modification.
